# Serum VEGF as a predictive marker of glycemic control and diabetic nephropathy in Chinese older adults with type 2 diabetes mellitus

**DOI:** 10.3389/fendo.2023.1274025

**Published:** 2023-11-22

**Authors:** Yanyan Jiang, Jianhua Li, Juan Zhang, Sufang Chen

**Affiliations:** ^1^ Department of Geriatric Endocrinology, The First Affiliated Hospital of Zhengzhou University, Zhengzhou, China; ^2^ Department of Emergency Medicine, The First Affiliated Hospital of Zhengzhou University, Zhengzhou, China; ^3^ Institute of Monogenic Disease, School of Medicine, Huanghuai University, Zhumadian, China

**Keywords:** vascular endothelial growth factor, glycemic control, diabetic nephropathy, type 2 diabetes mellitus, older adults

## Abstract

**Objectives:**

Recent researches have demonstrated good correlation between vascular endothelial growth factor (VEGF) and diabetic nephropathy (DN); however, this relationship seems less clear-cut when VEGF was measured in blood samples. We tended to explore the possible association between serum VEGF and glycemic control and diabetic nephropathy severity in Chinese older adults with type 2 diabetes mellitus (T2DM).

**Materials and methods:**

This study retrospectively enrolled 595 older T2DM adults at random. Participants were clinically grouped across the urine albumin-to-creatinine ratio (UACR) and the HbA1c tertiles by genders. Linear regressions were performed for the correlation of VEGF with HbA1c and UACR and binary logistic regressions for the odds of DN after adjusting for confounders. The receiver operating characteristic (ROC) curves were conducted for the predictive value of VEGF for DN.

**Results:**

Both males and females with DN exhibited higher VEGF levels than non-DN (*P* < 0.001). Furthermore, a positive correlation of VEGF with UACR and HbA1c was presented regardless of adjusting confounding factors (*P* < 0.001). Serum VEGF level and fasting plasma glucose (FPG) were independent risk factors of DN in older adults of both genders (*P* < 0.05), while the risk prediction of DN by HbA1c only reflected in female patients (*P* < 0.05). The ROC curve of VEGF for DN had the area under curve (AUC) of 0.819 for males and 0.793 for females, indicating the clinical value of serum VEGF as a predictive biomarker.

**Conclusions:**

Serum VEGF was strongly associated with UACR and HbA1c in both genders, and could be regarded as a predictive biomarker for glycemic control and diabetic nephropathy in older adults with T2DM.

## 1 Introduction

Human ageing is accompanied by a progressive decline in kidney function. Elderly subjects with type 2 diabetes mellitus (T2DM) are at a greater risk of diabetic nephropathy (DN) (1). The prevalence of diabetes is also higher in older adults. Nearly 60% of patients with T2DM are adults aged ≥60 years, with the highest prevalence in the age range of 75–79 years (2). Furthermore, 30% of patients with T2DM are associated with DN, which progresses to end-stage renal disease (ERSD) with increasing age and duration of T2DM and requires dialysis, thereby significantly burdening the public health system (3, 4). Therefore, early screening and diagnosis of DN is necessary for timely intervention that can significantly delay the progression of DN in older subjects with T2DM.

Vascular endothelial growth factor (VEGF), a major regulator of vascular permeability and angiogenesis, plays a significant role in diabetic albuminuria and in the pathogenetic mechanisms underlying diabetic nephropathy (5). Furthermore, VEGF is associated with adverse effects in subjects with DN and protective effects in the non-DN individuals (6). Therefore, tight regulation of VEGF levels is critical for the maintenance of glomerular filtration and renal health. In the experimental animal models of diabetes, VEGF is significantly elevated in the kidney tissues and blockade of VEGF signaling ameliorates diabetic albuminuria (7, 8). The correlation between VEGF and human diabetic nephropathy is controversial with many studies reporting contradictory findings (5, 6). Several clinical studies have reported that elevated serum VEGF levels are associated with the development of DN (9–12). However, other studies have shown absence of any association or a negative relationship between circulating VEGF levels and diabetic albuminuria (13–15).

Currently, the worldwide prevalence of DN in older adults is gradually increasing with a higher proportion of older individuals developing uremia (4). Therefore, there is greater emphasis in determining the role of VEGF in early DN and its potential as a diagnostic and prognostic biomarker for DN (16–18). Thus, in this study, we investigated the association of VEGF with glycemic control and DN in elderly subjects with T2DM. Furthermore, we estimated the cut-off value for serum VEGF in the early detection of DN among elderly subjects with T2DM.

## 2 Materials and methods

### 2.1 Study participants

This retrospective study recruited elderly subjects with T2DM who were treated as inpatients at the Department of Geriatric Endocrinology, the First Affiliated Hospital of Zhengzhou University from June 2021 to June 2023. These participants were diagnosed with diabetes according to the standard diagnostic criteria specified by the American Diabetes Association (ADA) guidelines in 2021 (19).

**Figure 1:**
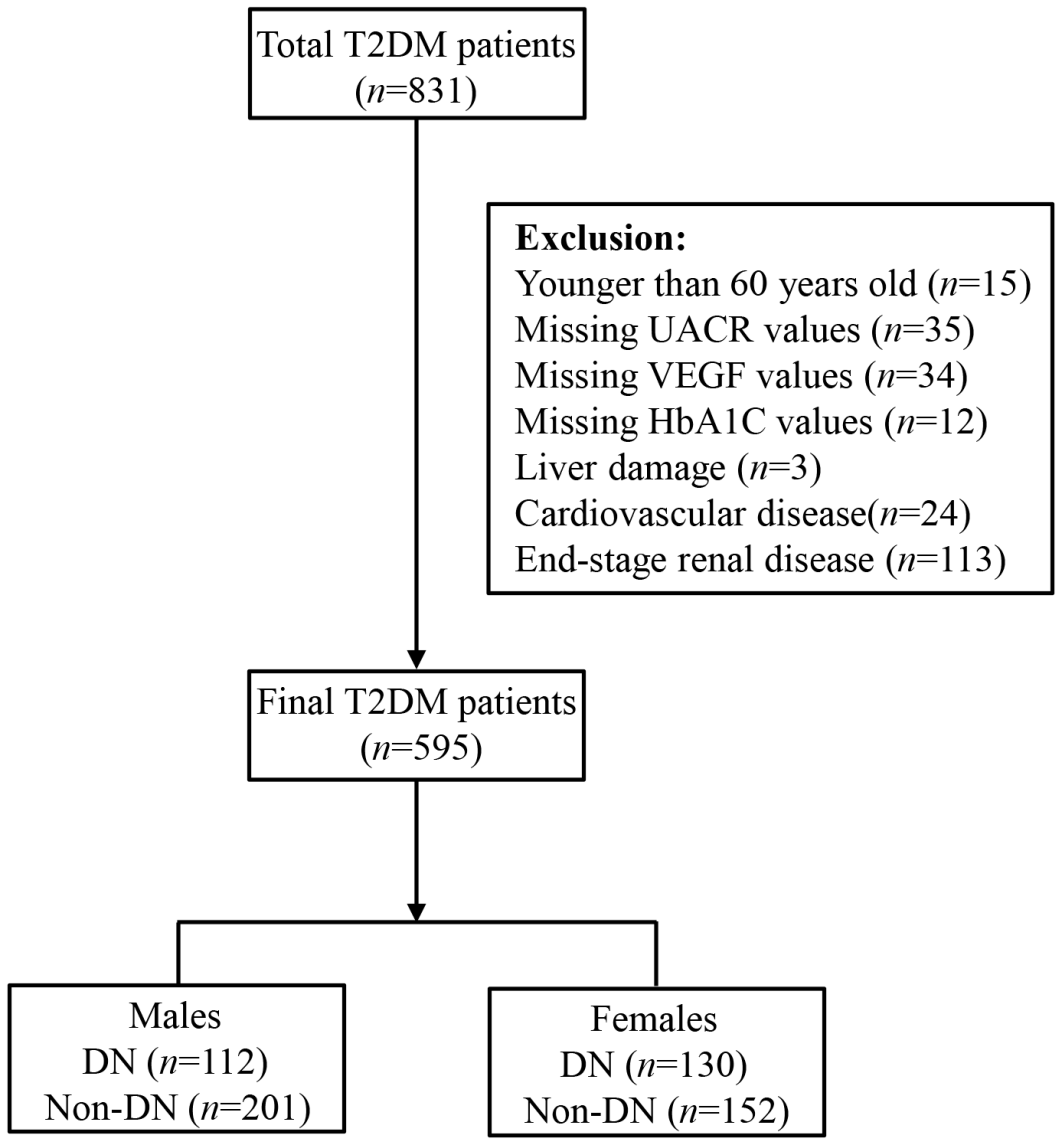
Flowchart of study participants.

We then excluded (1) subjects younger than 60 years (n = 15), and (2) subjects missing UACR (n = 35), VEGF (n = 34), and/or HbA1c (n =12) values, and (3) subjects with liver damage (n = 3), coronary heart disease (n = 24), and/or ESRD (n = 113). Finally, we included 595 study subjects, including 313 males and 282 females in this investigation, and categorized them into DN (n = 242) and non-DN groups (n = 353) (**Figure 1**). DN was defined according to the diagnostic criteria recommended by the Kidney Disease: Improving Global Outcomes (KDIGO) Diabetes Work Group (20). This study was performed according to the Declaration of Helsinki principles and was approved by the Ethics committee of the First Affiliated Hospital of Zhengzhou University. All the participants voluntarily signed informed consent before participating in this study.

### 2.2 Clinical data

We collected clinical data from the medical records, including gender, age, diabetes duration, height, weight, and blood pressure. The body mass index (BMI, kg/m^2^) was calculated by dividing the weight (kg) by the squared value of the height (m). The venous blood samples were collected from the included study subjects after overnight fasting, and the biochemical parameters, including total cholesterol (TC), triglycerides (TG), high-density lipoprotein (HDL) cholesterol, and low-density lipoprotein (LDL) cholesterol were analyzed using an automated biochemical analyzer (Hitachi 7600-020, Japan). The venous blood glucose levels were estimated using the glucose oxidase method. The apolipoprotein levels were estimated by immune transmission turbidimetry. HbA1c levels were estimated using standard high-performance liquid chromatography (HPLC, Bio-Rad, Hercules, CA, USA). We also collected the first urine sample in the morning and analyzed the urinary albumin creatinine ratio (UACR).

### 2.3 ELISA assay

Fresh blood samples were collected in polymer gel chemistry tubes and kept at room temperature for 20 mins. Then, the blood samples were centrifuged at 3000 rpm for 5 mins and the serum was collected. The serum VEGF levels were estimated using a VEGF-specific ELISA kit (Beijing Jianping Venus Biotechnology Co., Ltd., Beijing, China) according to the manufacturer’s instructions.

### 2.4 Statistical analysis

Statistical analysis was performed using the SPSS statistical software 28.0 (IBM Corp, USA). The graphs were generated using the GraphPad Prism 8.0 software (GraphPad Software, California, USA). Normality of data was analyzed using the Shapiro-Wilk test. The data were presented as mean ± standard deviation (SD), median (interquartile range; IQR), or number (percentage). Non-normally distributed data were logarithmically transformed before analysis. Student t test and Mann-Whitney U test were used to estimate the statistical differences between two groups. The analysis of covariance (ANCOVA) was used to compare the natural log (Ln) of serum VEGF levels across the tertiles of HbA1c and clinical groups based on UACR after adjusting for age and BMI. Chi-square test (χ^2^) was used to compare categorical variables. Linear regression analysis was used to analyze the correlation of VEGF with HbA1c and UACR. Binary logistic regression analysis was used to identify independent risk factors of DN based on the odds ratio after adjusting for potential confounding factors. The confounding factors were defined as variables with statistical differences in **Table 1**. Finally, the receiver‐operating characteristic (ROC) curves were generated to estimate the clinical performance of serum VEGF for predicting the occurrence of T2DM-induced nephropathy based on gender. The Youden Index was calculated as sensitivity + specificity – 1, and used to determine the cut-off values. *P <*0.05 was considered as statistically significant.

**Table 1:** Baseline characteristics of older adults with T2DM.

Variables	Males			Females		
	DN	Non-DN	*P* value	DN	Non-DN	*P* value
N (%)	112 (35.8%)	201 (64.2%)		130 (46.1%)	152 (53.9%)	
Age (year)	69.46 ± 0.48	70.15 ± 0.42	0.292	70.19 ± 0.49	69.57 ± 0.49	0.361
Duration (year)	7.00 (1.00, 13.00)	3.00 (0.46, 8.00)	0.000	7.50 (3.00, 13.25)	5.00 (1.00, 10.00)	0.009
BMI (kg/m^2^)	25.65 ± 0.45	25.54 ± 0.27	0.829	24.81 ± 0.32	25.16 ± 0.32	0.447
DBP (mmHg)	87.92 ± 1.24	84.69 ± 0.90	0.034	84.29 ± 1.32	81.10 ± 1.06	0.057
SBP (mmHg)	142.21 ± 1.99	131.75 ± 1.27	0.000	141.86 ± 2.23	133.52 ± 1.65	0.003
Diabetic retinopathy, N (%)	60 (53.57%)	55 (27.36%)	0.000	54 (41.54%)	56 (36.84%)	0.420
UACR (mg/g)	100.03 (35.63, 728.50)	7.90(1.90, 19.45)	0.000	119.03 (47.80, 645.08)	4.16 (1.43, 10.98)	0.000
VEGF (pg/mL)	253.22 (183.19, 328.56)	140.14 (105.07, 178.40)	0.000	180.32 (132.91, 275.19)	87.09 (62.75, 141.83)	0.000
HbA1c (%)	9.88 ± 0.23	10.30 ± 0.17	0.193	10.81 ± 0.23	9.93 ± 0.20	0.004
FPG (mmol/L)	7.82 (6.63, 10.26)	7.59 (6.43, 8.72)	0.059	8.42 (7.25, 9.90)	7.71 (6.69, 9.10)	0.009
TC (mmol/L)	4.49 (3.61, 5.33)	4.54 (3.73, 5.26)	0.669	4.91 (4.00, 5.89)	4.74 (3.67, 5.51)	0.041
TG (mmol/L)	1.43 (0.94, 2.34)	1.65 (1.00, 2.61)	0.113	1.46 (1.00, 2.63)	1.50 (0.99, 2.12)	0.091
HDL (mmol/L)	0.95 (0.82, 1.19)	1.01 (0.85, 1.18)	0.437	1.10 (0.91, 1.31)	1.11 (0.97, 1.34)	0.580
LDL (mmol/L)	2.57 ± 0.08	2.63 ± 0.07	0.605	2.84 ± 0.10	2.74 ± 0.08	0.436
apoA (g/L)	1.45 ± 0.03	1.44 ± 0.02	0.823	1.54 ± 0.31	1.58 ± 0.02	0.253
apoB (g/L)	0.86 ± 0.03	0.86 ± 0.02	0.997	0.95 ± 0.03	0.85 ± 0.02	0.009
ALT (U/L)	18.00 (12.00, 30.00)	18.00 (14.00, 29.00)	0.799	14.00 (9.00, 24.00)	15.00 (11.00, 22.75)	0.301
AST (U/L)	19.50 (15.25, 25.75)	18.00 (15.00, 22.00)	0.151	18.00 (15.00, 25.00)	18.00 (15.00, 24.75)	0.721
ALP (U/L)	77.00 (64.00, 94.00)	79.00 (64.00, 94.00)	0.945	89.00 (70.00, 109.00)	79.00 (64.00, 94.00)	0.006
GGT (U/L)	29.00 (19.00, 42.75)	28.00 (20.00, 43.00)	0.979	20.00 (15.75, 32.00)	21.50 (16.00, 32.00)	0.614
BUN (mmol/L)	5.70 (4.72, 7.38)	5.30 (4.30, 6.60)	0.012	5.40 (4.30, 7.42)	4.75 (3.78, 5.50)	0.000
Cr (umol/L)	71.50 (61.00, 88.75)	66.00 (58.00, 77.00)	0.000	57.00 (48.00, 78.00)	54.00 (46.25,63.75)	0.019
eGFR (mL/min/1.73m2)	116.67 (88.57, 139.70)	128.79 (107.69, 142.13)	0.001	140.86 (107.97, 152.28)	145.70 (134.86, 153.46)	0.009
Uric acid (mmol/L)	297.50 (226.00, 366.75)	278.00 (225.00, 331.50)	0.179	245.00 (197.75, 299.00)	241.00 (186.50, 286.75)	0.388

Data were described as mean ± standard deviation (SD), median and interquartile range (IQR), number (%), as appropriate.

Duration, diabetes duration; BMI, body mass index; UACR, urine albumin/creatinine ratio; TC, total cholesterol; TG, triglycerides; HDL, high-density lipoprotein cholesterol; LDL, low-density lipoprotein cholesterol; VEGF, vascular endothelial growth factor; FPG, fasting plasma glucose; HbA1c, glycated hemoglobin; ALT, alanine aminotransferase; AST, aspartate aminotransferase; ALP, alkaline phosphatase; GGT, glutamyltranspeptidase; apoA, apolipoprotein A; apoB, apolipoprotein B; BUN, blood urea nitrogen; Cr, creatinine; eGFR, estimated glomerular filtration rate.

## 3 Results

### 3.1 Demographic information and clinical characteristics of the study subjects

This study included 282 females and 313 males. The prevalence of DN was 46.1% among women and 35.8% among men. The baseline demographic, clinical, and laboratory profiles of the study subjects are shown in **Table 1**. The median VEGF levels were 253.22 pg/mL (183.19, 328.56) and 140.14 pg/mL (105.07, 178.40) for the male DN and non-DN groups, respectively, and 180.32 pg/mL (132.91, 275.19) and 87.09 pg/mL (62.75, 141.83) for the female DN and non-DN groups, respectively (all *P* < 0.001). Among males, the DN group subjects showed higher BP (*P* < 0.05), longer course of diabetes (*P* < 0.001), higher frequency of diabetic retinopathy (*P* < 0.05), worse renal dysfunction based on higher BUN levels (*P* < 0.05), higher creatinine levels (*P* < 0.001), lower eGFR values (*P* < 0.01), and higher UACR (*P* < 0.001), and higher serum VEGF levels (*P* < 0.001) compared with the non-DN subjects. Among females, the DN group subjects showed higher SBP (*P* < 0.01), longer duration of diabetes (*P* < 0.01), higher BUN levels (*P* < 0.001), higher creatinine levels (*P* < 0.05), higher UACR (*P* < 0.001), lower eGFR (*P* < 0.01), higher serum VEGF levels (*P* < 0.001) and worse glycemic control characterized by higher HbA1c (*P* < 0.01) and higher fasting plasma glucose (FPG) levels (*P* < 0.01) compared with the non-DN subjects.

### 3.2 Association of serum VEGF with HbA1c and UACR

**Figure 2:**
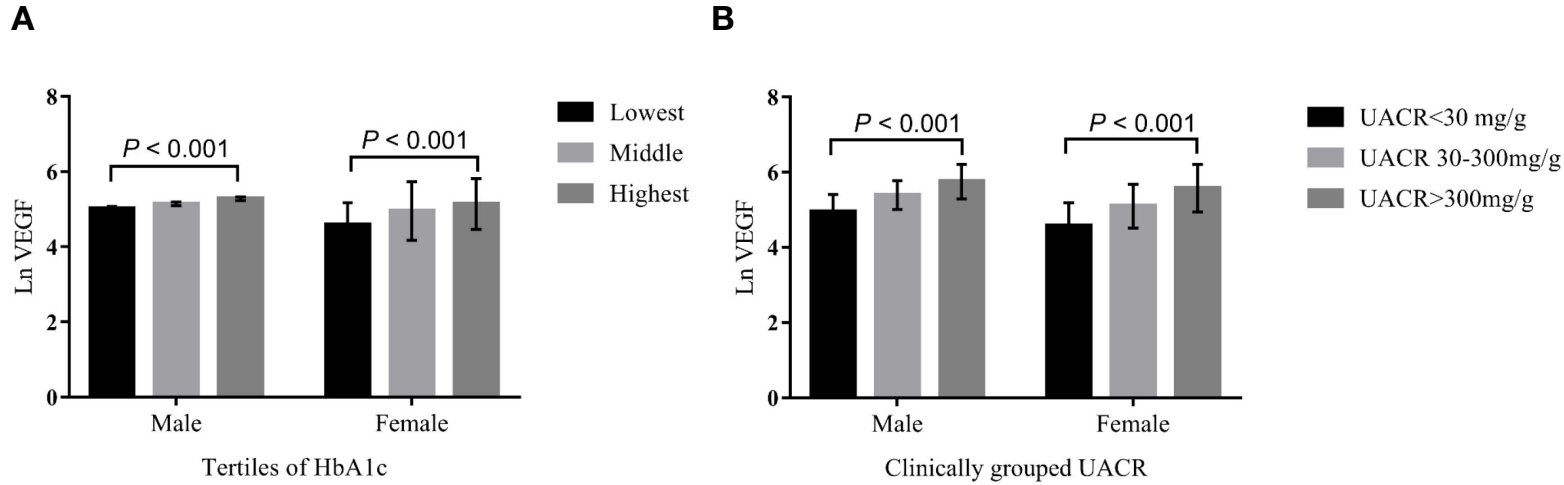
The VEGF levels across the HbA1C tertiles **(A)**, and clinically grouped UACR **(B)**. ANCOVA was performed on Ln transformed VEGF, and age and BMI were adjusted. HbA1c (%) were divided into tertiles by genders, male: lowest tertile 4.54-9.00; middle tertile 9.06–11.08; highest tertile11.09-17.69; Female: lowest tertile 4.92-9.03; middle tertile 9.07-11.37; highest tertile 11.43-19.08. Based on the UACR, the patients were divided into three groups by genders: UACR < 30 mg/g, UACR 30–300 mg/g and UACR > 300 mg/g.

Linear regression analysis demonstrated that serum VEGF levels were positively associated with HbA1c and UACR in both males and females, before and after adjustment of potential confounding factors (all *P* < 0.001) (**Table 2**). Furthermore, Ln (VEGF) showed an increasing trend with elevated HbA1c levels and UACR after adjusting for age and BMI in both the genders (all *P* < 0.001) (**Figure 2**). Stratifying by HbA1c tertiles, there was a significant increase in Ln VEGF from the lowest vs. the highest tertile, while the middle tertile did not show a significant difference with the lowest tertile in males, and this was visually depicted in **Figure 2**.

**Table 2:** Linear regression analysis for the association between VEGF level and indices.

Variables	Ln VEGF (Male)		Ln VEGF (Female)	
	Standardized β (*t*)	*P* value	Standardized β (*t*)	*P* value
HbA1c (%)				
Model1	0.248 (4.511)	0.000	0.296 (5.188)	0.000
Model2	0.291 (4.985)	0.000	0.286 (4.787)	0.000
Model3	0.307 (5.379)	0.000	0.282 (4.725)	0.000
UACR (mg/g)				
Model1	0.472 (9.438)	0.000	0.211 (3.603)	0.000
Model2	0.467 (9.138)	0.000	0.195 (3.330)	0.001
Model3	0.456 (8.215)	0.000	0.160 (2.632)	0.009

Data are expressed as standardized β coefficients and t. VEGF was Ln transformed to correct skewed distribution.

Model 1: Crude model.

Model 2: adjusted for SBP, Duration, TC, ALP.

Model 3: adjusted for SBP, Duration, TC, ALP, BUN, and Cr.

### 3.3 Risk factors for DN in older adults with T2DM

**Figure 3:**
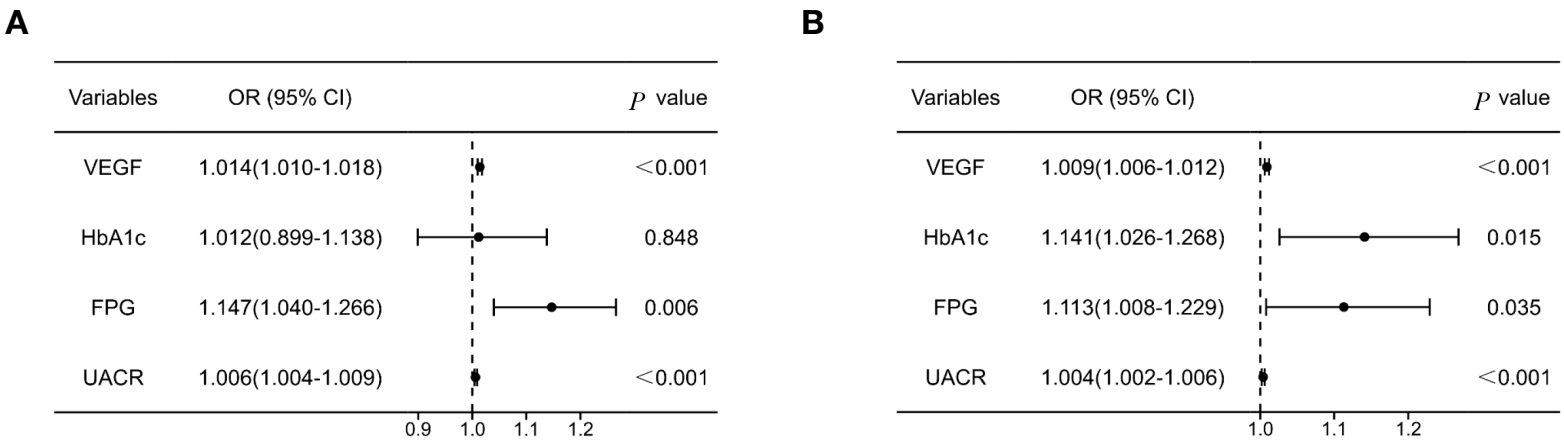
Forest plot of logistic regression model investigating risk factors for DN in males **(A)** and females **(B)** with T2DM. Model was adjusted for SBP, Duration, TC, ALP, BUN, Cr. SBP, systolic pressure; TC, total cholesterol; ALP, alkaline phosphatase; BUN, blood urea nitrogen; Cr, creatinine.

Binary logistic regression analysis was performed to identify the risk factors for DN in older T2DM individuals. In males, ORs for the development of DN were 1.014 (95% CI 1.010, 1.018), 1.147 (95% CI 1.040, 1.266) and 1.006 (95% CI 1.004, 1.009) for every 1 SD increase in serum VEGF, FPG, and UACR, respectively, after adjusting for the confounding factors (all *P* < 0.05). In the females, ORs for the development of DN were 1.009 (95% CI 1.006, 1.012), 1.113 (95% CI 1.008, 1.229) and 1.004 (95% CI 1.002, 1.006) for every 1 SD increase in serum VEGF, FPG, and UACR, respectively, after adjusting for the confounding factors (all *P* < 0.05) (**Figure 3**). The correlation between VEGF, FPG, UACR and DN were similar in both genders. Furthermore, HbA1c levels showed positive correlation with DN in the female subjects (*P* < 0.05) (**Figure 3**).

### 3.4 Predictive value of serum VEGF for DN

**Figure 4:**
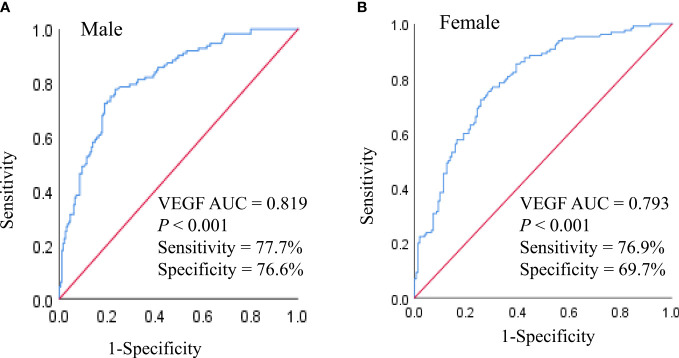
ROC analysis of VEGF to indicate DN in older males **(A)** and females **(B)** with T2DM. AUC, Area under the curve.

Finally, we performed ROC curve analysis to determine the predictive performance of serum VEGF levels for DN in older subjects with T2DM. In the male subjects, the AUC value for serum VEGF was 0.819 (95% CI 0.772 - 0.860) (*P* < 0.001) at the cut-off value of 179.40 pg/mL; in the female subjects, the AUC value for serum VEGF was 0.793 (95% CI 0.741–0.839) (*P* < 0.001) at the cut-off value of 131.57 pg/mL (**Figure 4**). This demonstrated that serum VEGF was a promising predictive biomarker for DN in older adults with T2DM regardless of the gender.

## 4 Discussion

This was the first study to investigate the correlation between serum VEGF levels and DN in Chinese T2DM adults older than 60 years. Systemic vascular endothelial homeostasis disorder develops before the signs of microvascular or macrovascular complications are evident in patients with diabetes (21, 22). VEGF plays a vital role in endothelial dysfunction and is associated with both DN and proliferative retinopathy (23). Multiple studies have reported a positive correlation between serum VEGF levels and DN in patients with type 1 and type 2 diabetes (9–12). Our study demonstrated that the serum VEGF was a risk factor for DN in older adults with T2DM and showed positive association with UACR and HbA1c (**Table 2**; **Figures 2**, **3**). This relationship was independent of SBP, duration of diabetes, TC, ALP, BUN, and creatinine. Yang et al. evaluated 107 T2DM patients with an average age of 49.27 ± 4.26 years and showed an increasing trend for the serum VEGF levels between the normal urinary protein, microproteinuria, and the massive proteinuria groups; moreover, changes in the serum VEGF levels were positively associated with the progression of DN (*r* = 0.518, *P <* 0.001) (12). The results of our study concurred with the findings of Yang et al.

However, the relationship between serum VEGF and DN is controversial. Multiple studies have reported that serum VEGF levels do not associated with progression of DN (13, 15). These differences between studies may be due to variations in the race of the study subjects, sample sizes, or VEGF detection specimens (plasma versus serum (13, 15). In the serum, a large amount of VEGF protein is derived from the activated platelets (10). Hanefeld et al. also confirmed that VEGF-A in the serum was derived mostly from the platelets and better reflected the glycemic burden than the plasma VEGF-A levels (10). Schlingemann et al. reported that β-thromboglobulin, a biomarker for *in vivo* platelet activation, showed positive correlation with proteinuria, thereby confirming the relationship between DN and increased *in vivo* platelet activation (24). VEGF derived from the activated platelets and the podocytes mediates endothelial dysfunction and glomerular damage in patients with diabetes, thereby contributing to the progression of DN (24–26).

Kakizawa et al. evaluated 45 Japanese diabetic individuals aged 26-79 years and did not observe any significant changes in the VEGF levels at various degrees of proteinuria (27). However, this may have been caused by a large age range of the subjects in this study. Our study focused on the elderly subjects above 60 years of age. Moreover, we used a large sample size to achieve consistent data regarding the relationship between VEGF and DN. Thus, our study provides strong evidence for serum VEGF being a promising predictor of DN risk in elderly subjects with T2DM.

Our data also suggested that DN patients with elevated serum VEGF levels were more prone to retinopathy than the non-DN patients (**Table 1**). This was consistent with previous reports that demonstrated significantly higher serum VEGF levels in diabetic patients with proliferative retinopathy (28).

VEGF-A is a member of the VEGF family of proteins and regulates vascular permeability and angiogenesis (29). VEGF-A is often be referred to as VEGF. The other members of the VEGF family of proteins are VEGF-B, VEGF-C, VEGF-D, VEGF-E, and placental growth factor (PlGF) (30). Mechanistically, podocyte-derived VEGF-A binds to VEGF receptor-2, which is expressed on the surface of the glomerular endothelial cells, and mediates changes in the vascular permeability and vascular endothelial damage in the kidneys (6). Therefore, interventions that suppress VEGFA-VEGFR2 signaling delay the onset of early kidney disease in patients with diabetes (6). VEGF-B is another member of the VEGF family of protein with weak angiogenic effects. VEGF-B is associated with renal dysfunction in patients with T2DM (31, 32). VEGF-C overexpression reduces glomerular permeability and protects against altered VEGF receptor expression, thereby improving glomerular and endothelial barrier functions (33). Therefore, future investigations are necessary to determine the roles of specific VEGF subtypes in the occurrence and progression of DN among the elderly subjects.

Kakizawa et al. reported that plasma VEGF levels were positively associated with FPG and HbA1c; moreover, plasma VEGF levels decreased after comprehensive hypoglycemic treatment with insulin or oral hypoglycemic agents (27). Our results also showed that the serum VEGF levels were associated with glycemic control (HbA1c) in the older adults with T2DM (**Table 2**). Therefore, VEGF plays a significant role in diabetes. However, analysis of the tertiles of HbA1c against Ln (VEGF) in male subjects showed significant differences only between the lowest tertile and the highest tertile of HbA1c, but the Ln (VEGF) estimates did not show statistical differences between subjects in the lowest and the medium tertiles of HbA1c (**Figure 2**). This indicated presence of gender-related differences in the relationship between serum VEGF and HbA1c. Kajiwara et al. investigated sex differences in the decline of renal function among Japanese patients with T2DM and reported significant correlation between HbA1c and eGFR decline only in females (34). This may be attributed to poorer metabolic control in females because women experience greater hormonal fluctuations and physical changes than men during their lifetime (35). The elevated VEGF concentration is a sign of poor blood glucose control. Therefore, serum VEGF levels show a better clinical value for predicting the risk of DN. VEGF expression is upregulated in multiple cell types and is indicative of poor glycemic control in the diabetic patients and animal models of diabetes (10, 24, 36). The narrow physiological range of VEGF-A is not only critical for maintaining the optimal kidney function, but also plays a significant role in maintaining the homeostasis and functions of the pancreatic islets in adults 29). Mice with specific down-regulation of VEGF-A in the insulin-producing β cells demonstrated significant reduction in the islet microvascular density and glucose-stimulated insulin secretion (37, 38), whereas overexpression of VEGFA impaired glucose tolerance and β cell mass (39, 40). Therefore, we speculated that hyperglycemia in the older adults with T2DM upregulated VEGF expression. Sustained overexpression of VEGF worsens glycemic control through its effects on the vascular endothelial cells of the islets and the glomerulus, thereby promoting the development of diabetic nephropathy. However, elevated circulating levels of VEGF are not sufficient to determine the corresponding changes in the islet β cells and the glomerulus. Therefore, determination of local VEGF levels may be necessary to establish and confirm the association between higher VEGF levels and dysfunction of the glomerulus and the pancreatic β cells.

The present study has several limitations. Firstly, this was a retrospective observational study. Therefore, we did not analyze the causal relationship between serum VEGF levels and UACR. Moreover, reverse causality cannot be excluded. Secondly, higher VEGF levels may be derived from platelet activation during blood collection and may be a confounding factor in this study. Thirdly, the influence of hypoglycemic treatment on the blood glucose control was not analyzed. Finally, this was a single center study. Furthermore, we did not perform any follow-up of the patients. Therefore, in the future, multi-center, large-cohort prospective studies are necessary to confirm our findings.

In conclusion, this study demonstrated that elevated serum VEGF levels were associated with poor glycemic control and progression of DN in older adults with T2DM. Furthermore, serum VEGF is a promising biomarker for the early detection of DN in the diabetic patients and can be used for treating susceptible individuals with effective therapies for better glycemic control to prevent DN and its progression.

## Data availability statement

The original contributions presented in the study are included in the article/supplementary material. Further inquiries can be directed to the corresponding author.

## Ethics statement

The studies involving humans were approved by the First Affiliated Hospital of Zhengzhou University Ethics Review Committee. The studies were conducted in accordance with the local legislation and institutional requirements. The participants provided their written informed consent to participate in this study.

## Author contributions

YJ: Conceptualization, Data curation, Formal Analysis, Funding acquisition, Methodology, Project administration, Validation, Visualization, Writing – original draft. JL: Conceptualization, Data curation, Formal Analysis, Investigation, Methodology, Project administration, Software, Validation, Visualization, Writing – original draft. JZ: Conceptualization, Data curation, Formal Analysis, Funding acquisition, Investigation, Methodology, Project administration, Resources, Software, Validation, Visualization, Writing – original draft. SC: Writing – review & editing.
